# Knowledge of and preferred sources of assistance for physical activity in a sample of urban Indigenous Australians

**DOI:** 10.1186/1479-5868-5-22

**Published:** 2008-04-28

**Authors:** Alison L Marshall, Julian Hunt, David Jenkins

**Affiliations:** 1 School of Public Health, Queensland University of Technology, Brisbane, Australia; 2 School of Human Movement Studies, University of Queensland, Brisbane, Australia

## Abstract

**Background:**

To examine urban Indigenous Australians' knowledge of the current Physical Activity Guidelines (PAG) and identify their preferred sources of assistance or advice regarding physical activity.

**Method:**

Self-completed questionnaire data were collected from 194 participants; the questionnaires sought information on standard demographics including an assessment of their perceived physical activity level relative to peers. Outcome measures were agreement with five statements from the current PAG and indicators of preferred sources of assistance or advice regarding physical activity.

**Results:**

Most participants demonstrated excellent knowledge of the current PAG, with 92% to 88% of participants agreeing with the statements. Significantly more older participants (> 44 years) identified that 'blocks of 10 minutes of activity are OK' compared to younger participants (aged 18–44 years: 60%; *X*^2 ^= 6.23; *p *= .04). Significantly more higher educated participants agreed (96%) that 'brisk walking for half an hour most days was good for health' compared to the less educated participants (85%; *X*^2 ^= 8.08; *p *= .02). The most preferred source of physical activity advice identified by men was the GP/health professional (62% vs. 53%; men and women respectively, NS), while for women it was a group to be active with (60% vs. 42%; women and men respectively; X2 = 6.09; p = .01).

**Conclusion:**

Urban Indigenous Australians have similar levels of knowledge regarding the PAG to non-Indigenous Australians. However, the option of accumulating 10-minute activity bouts needs to be better communicated to younger Indigenous people. Most participants expressed a preference for advice about physical activity to be delivered via health professionals, and groups to be active with. Indigenous and age-specific resources that promote the unique aspects of the current PAG (e.g., that vigorous exercise is not essential for health and blocks of 10 minutes of activity are OK) should be developed and disseminated.

## Background

In 2001, Australia's Indigenous population was estimated to be 458,520 [1]. A generally negative picture is often reported regarding the past and present state of Indigenous Australians' health. Data consistently show that Indigenous Australians have a significantly lower life expectancy and are more likely to experience disability and reduced quality of life due to ill health compared to non-Indigenous Australians [2]. Death from ischemic heart disease in Indigenous Australians is twice the rate found in the non-Indigenous population, and six to eight times higher in those aged 25–65 years [3]. Many Indigenous people also suffer from impaired glucose metabolism; the age-standardised prevalence of impaired glucose metabolism was recently found to be over three times the rate reported for non-Indigenous Australians [4].

Interestingly most Indigenous Australians live in urban locations (70%), yet most attention is paid to rural and remote Indigenous communities where the ratio of Indigenous people to non-Indigenous people is higher [5]. However, the burden of cardiovascular disease risk factors and ill health is equally high among both urban and rural/remote Aboriginal communities [6,7].

Physical activity is an independent risk factor for both heart disease and diabetes, and given the high prevalence of these chronic diseases among Indigenous Australians it is surprising that little is known about the physical activity patterns of Indigenous Australians. In fact no data specific to Indigenous Australians' physical activity patterns, knowledge or intentions were included in the most recent reports on physical activity patterns of Australian adults [8,9].

To date, many health interventions offered to Indigenous people have focused on changing diet and nutrition. Authors of a recent evaluation of community store intervention and education program reported that interventions targeting nutritional factors alone are unlikely to have a significant effect on reducing the rising prevalence of diabetes and obesity, and that the role of physical activity in improving metabolic fitness should be emphasised [10]. However, it is difficult to develop such programs when Indigenous peoples' knowledge of current physical activity messages is yet to be evaluated [11].

The primary aim of this research was to examine urban Indigenous Australians' knowledge of messages consistent with the current Physical Activity Guidelines (PAG) [12], to inform the development of appropriate programs to promote physical activity with this population group. A secondary aim was to assess urban Indigenous Australians' preferred sources of advice or assistance for increasing physical activity, so that further investigations and/or programs can be developed to build the capacity of preferred providers, and/or delivery modes and mechanisms.

## Methods

### Participant recruitment

Negotiating top down support from Indigenous community leaders was a vital first step in seeking approval for this study and consequently identifying potential participants. Available resources and consultation suggested it was not appropriate to randomly select study participants, thus participants were recruited via convenience sampling through various urban Indigenous community organisations, groups and newspaper advertisements. Similar recruitment methods have been reported in other Indigenous health research [6]. Eligible participants; were aged over 18 years, identified as Aboriginal and/or Torres Strait Islander, resided in Brisbane, and were able to read and speak English.

Potential participants were introduced to the purpose of the study and given an information sheet by trained Indigenous research assistants. Participants were required to give written informed consent before completing the study. Study procedures were endorsed by the Brisbane Southside Indigenous Public Health Forum and approved by the Human Research Ethics Committee at The University of Queensland.

### Data collection

Data were collected via a self-complete questionnaire; a method used in previous research with Indigenous adults [6,13]. The questionnaire included standard demographic items (Indigenous status, age, gender, height, weight, marital status, education, employment and whether they had children). Participants were also asked to rate their current physical activity level relative to other Indigenous people of the same age and sex on a 5-point scale: (1) much more active, (2) more active, (3) about the same, (4) less active, or (5) much less active. This question was used due to the absence of a valid and reliable measure of Indigenous Australians physical activity [14]. A similar question and item response categories have been used in previous national physical activity surveys, and these have demonstrated good concordance with identifying people who are meeting PAG (versus those who are not) [15].

Knowledge of the key messages associated with the PAG was assessed using items previously used to assess non-Indigenous Australians' physical activity knowledge [8,15,16]. Each participant was asked to rate their agreement (on a five-point scale from (1) 'strongly agree' through to (5) 'strongly disagree') with each of the following statements: (a) Taking the stairs at work or generally being active for at least 30 minutes each day is enough to improve your health; (b) half an hour of brisk walking on most days is enough to improve your health; (c) to improve your health, it is essential for you to do vigorous exercise for at least 20 minutes each time, three times a week; (d) exercise doesn't have to be done all at one time – blocks of ten minutes are OK, and (e) moderate exercises that increase your heart rate slightly can improve your health.

The participants' preferred sources for physical activity advice were assessed using the items from the Pilot Survey of the Fitness of Australians [17]. Participants could select more than one preference from a list of nine potential sources, including an option for 'other'.

### Data analysis

Data were entered into Microsoft EXCEL then exported into SPSS v14 for analysis; alpha of .05 was used to assess statistical significance. Demographic information relating to gender, age, height, weight, highest level of education, marital status, employment status and how many children lived at home were summarised using descriptive statistics. Body mass index (BMI: weight/height^2^) was calculated for those participants who reported height and weight.

Differences in the proportions of participants who agreed with the physical activity knowledge questions were assessed by gender, age group and education using chi square statistics. Age data were dichotomised into 18–44 years or over 45 years; education was categorised as < 12 years education and ≥ 12 years education. These are the same categories as those reported in the most recent national dataset [8]. Level of agreement with the physical activity knowledge questions was also compared between participants who perceived they were more/much more active than their peers and those who perceived they were less active.

The frequencies with which participants identified the various sources of assistance/advice for physical activity were calculated. Differences in preferred sources of assistance/advice between genders, age groups and education categories were assessed using chi square statistics.

### Feedback session

Two months following data collection, participants and the Indigenous leaders and community organisations that helped with recruitment were invited to a community forum where the findings of the study were discussed and permission to publish the data was granted.

## Results

### Participants

Approximately 80% of those invited to participate in this study gave written informed consent and provided data. The research assistants were instructed to accept an individual's right to refuse participation, and not seek a reason why they did not want to be involved (as that may have been perceived as intimidating and/or coercive). However, some reasons that were offered by those invited to participate but who declined, included: 'generally not interested', 'no time', and that they felt they 'had been researched enough'. Data were collected from 194 participants; 145 (75%) Aboriginals, 18 (9%) Torres Strait Islanders, 15 (8%) Aboriginal/Torres Strait Islanders, and 16 (8%) other. Seventy-eight participants (40%) were men and 116 (60%) were women. Further demographic details are shown in Table 1.

**Table 1:** Demographic profile of the urban Indigenous Australian participants (n = 194)

	**Total sample n = 194**	**Men n = 78**	**Women n = 116**
	**Mean (SD)**	**Mean (SD)**	**Mean (SD)**
Reported Height (cm)	169 (10)	177 (9)	164 (9)
Reported Weight (kg)	81 (21)	90 (18)	74 (20)
BMI (kg/m2)	28 (6)	29 (5)	28 (7)
	**n (%)**	**n (%)**	**n (%)**
**Indigenous Status**			
Aboriginal	145 (75)	56 (72)	89 (77)
Torres Strait Islander	18 (9)	9 (12)	9 (8)
Aboriginal & Torres Strait Islander	15 (8)	7 (9)	8 (7)
Other*	16 (8)	6 (8)	10 (9)
**Age**			
18 to 44 years	136 (70)	52 (67)	84 (72)
45 to 54 years	58 (30)	26 (33)	32 (28)
**Education**			
less than 12 years	58 (30.5)	22 (29)	36 (32)
≥ HSC/Year 12 or equivalent	132 (69.5)	55 (71)	77 (68)
**Married**	100 (52)	37 (48)	60 (52)
**Employed Fulltime**	139 (73)	46 (59)	93 (80)
**Have children living at home**	100 (52)	28 (36)	74 (64)
**Perceived activity level**			
Much more/more active	75 (39)	35 (45)	40 (34)
About the same	119 (61)	26 (33)	38 (33)
Less/much less active	55 (29)	17 (22)	38 (33)

* including South Sea Islander

For the total sample, 39% of participants reported they were 'more/much more active' than their peers (see Table 2). Fewer women perceived they were more active than their peers (34%) than men (45%). Interestingly, 8% of the women reported they were 'much less active' than other Indigenous women, while none of the men reported this.

**Table 2:** Urban Indigenous Australians knowledge the current National Physical Activity Guidelines (% agree with statement), including differences by gender, age, education and perceived activity level

		**Gender**			**Age category**			**Education Category**			**Physical activity level**		
	**Total** **N = 194**	**Men** **n = 78**	**Women** **n = 116**		**18–44 years** **n = 136**	≥ **45 years** **n = 105**		**<12 years** **n = 136**	≥ **12 years^¥^** **n = 105**		**More active** **n = 75**	**Same/less active** **n = 119**	
**Physical Activity statement**	**n (%)**	**n (%)**	**n (%)**	**X**^2 ^**(p)**	**n (%)**	**n (%)**	**X**^2 ^**(p)**	**n (%)**	**n (%)**	**X**^2 ^**(p)**	**n (%)**	**n (%)**	**X**^2 ^**(p)**
					
a. Generally being more active good for health	170 (88)	68 (87)	102 (88)	0.10 (.75)	116 (86)	54 (93)	2.26 (.32)	54 (93)	112 (86)	2.45 (.29)	64 (85)	106 (90)	2.19 (.33)
													
b. Brisk walk half an hour daily good for health	179 (92)	74 (95)	105 (91)	1.30 (.25)	124 (91)	55 (95)	2.19 (.33)	49 (85)	126 (96)	**8.08 (.02)**	68 (91)	111 (93)	0.58 (.75)
													
c. Vigorous exercise 3 times per week for 20 mins per session is essential for health	132 (68)	49 (64)	83 (72)	1.33 (.24)	90 (66)	42 (74)	1.31 (.52)	41 (70)	87 (66)	2.06 (.36)	53 (71)	79 (67)	0.33 (.85)
													
d. Blocks of 10 minutes of activity are OK	127 (66)	51 (66)	76 (66)	0.01 (.91)	82 (60)	45 (79)	**6.23 (.04)**	40 (69)	84 (64)	.61 (.74)	54 (72)	73 (62)	2.12 (.35)
													
e. Moderate exercise can improve your health	174 (90)	68 (87)	106 (91)	0.8 (.35)	122 (90)	52 (90)	.42 (.79)	53 (91)	117 (89)	.86 (.65)	67 (89)	102 (90)	2.20 (.33)

^¥ ^HSC or equivalent

### Knowledge of the current physical activity guidelines

Most participants demonstrated excellent knowledge of the current PAG, with between 92% and 88% of participants agreeing with correct statements (see Table 2). The lowest levels of agreement were recorded for the statements, that 'blocks of 10 minutes of activity are OK' (66%) and that 'vigorous exercise three times per week for 20 minutes per session is essential for health' (68%).

There were no statistically significant differences between genders in terms of agreement with the knowledge statements. However, there was a trend for more women to agree with the statement regarding the relationship between vigorous exercise and health (72% vs. 64%).

Significantly more of the older participants (> 44 years: 79%) agreed that 'blocks of 10 minutes of activity are OK' than younger participants (60%; *X*^2 ^= 6.23; *p *= .04). There was also a trend for more of the older participants to agree that vigorous exercise was essential for good health (74%) compared to the younger participants (66%).

Only one significant difference was observed between the two categories of reported education. Significantly more of the participants with higher education agreed (96%) that 'brisk walking for half an hour most days was good for health' compared to the less educated participants (85%; *X*^2 ^= 8.08; *p *= .02).

There were no significant differences in levels of agreement with the knowledge statements between those participants who perceived they were more/much more active than their peers and those who identified as being less active (see Table 2).

### Participants preferred sources of assistance for physical activity advice

**Figure 1:**
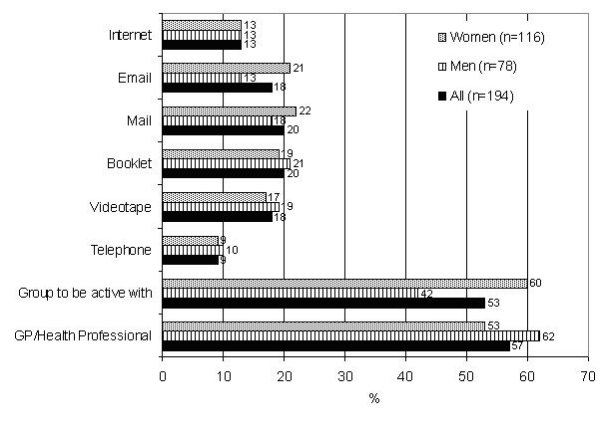
**Preferred sources of assistance or advice for physical activity in an urban Indigenous Australian sample (n = 194) including comparison of gender specific preferences.** NB: participants could select more than one preference.

Overall, most participants reported that they would prefer to receive advice or assistance with physical activity from a General Practitioner (GP) or health professional (57%) followed by a group they could be active with (53%) (see Figure 1). There were however, some interesting gender differences. The most frequently identified source of physical activity advice identified by men was the GP or health professional (62% vs. 53%; men and women respectively, NS), while for women it was a group to be active with (60% vs. 42%; men and women respectively; *X*^2 ^= 6.09; *p *= .01). Women were also more likely than men to identify a preference for advice to be delivered via mail (22%) or Email (21%), though these differences were not statistically significant. Few participants identified that they wanted to receive advice via the telephone (< 10%) or the Internet (13%).

There were no statistically significant differences by age group (data not shown) in terms of preferred sources of physical activity advice. However, more of those with ≥ 12 years of education identified a preference for receiving advice via Email compared to those who had less education (17% vs. 5% *X*^2 ^= 4.66; *p *= .03).

## Discussion

The aim of the present study was to describe urban Indigenous Australians' knowledge of the current PAG [12] and to identify preferences for advice or support relating to physical activity. Of the large number of urban Indigenous adults who participated in the research, more were women, many had completed high school and most were employed. Available data show that Indigenous Australians have higher rates of unemployment, tend to leave school at an earlier age and are less likely to gain post-secondary qualifications compared to non-Indigenous Australians [1]. However, most research conducted with Indigenous Australians carries the burden of overrepresentation in these groups [6]. Even with these limitations acknowledged, the present data advance our understanding of Indigenous Australians' knowledge of physical activity messages and their preferred sources of advice or assistance about physical activity.

The findings of this study are consistent with those reported by Armstrong et al. [8] and Bauman et al. [16] who evaluated knowledge of physical activity guidelines with non-Indigenous participants. Similar to the findings with non-Indigenous Australians, the large majority of the present population identified that 'brisk walking on most days', 'generally being more active' and exercising at a 'moderate intensity' are all good for health.

Also in agreement with the findings from the non-Indigenous survey (Armstrong et al.[8] and Bauman et al.[16]) was the that two thirds of the present participants believed that 'vigorous exercise is essential for health'. Moreover, one in three of the present Indigenous participants (compared to one in four non-Indigenous Australians) did not recognise that blocks of 10 minutes of activity can be beneficial for health. These findings are interesting because the current PAG's clearly suggest that vigorous exercise is not necessary for health benefits and that activity may be accumulated throughout the day in 10 minute bouts [12]. Collectively these findings suggest that these particular messages need to be better articulated. Moreover, the present research has shown that this may need to be relayed more effectively to younger Indigenous people.

This study also aimed to establish urban Indigenous peoples' preferred sources of advice and assistance for physical activity. Similar data have been reported by Booth et al. [17] from a large randomly selected sample (n = 2,298), and by Marshall et al. [18] from 797 responders to an online questionnaire. The trend across all these studies was a higher preference for advice or assistance from health professionals and via groups in which they could be active with (e.g., a walking group). Fewer participants in each study reported a preference for assistance or advice via the telephone, Internet, Email, books or videotape. Comparisons between the studies revealed that few participants (< 10%) wished to receive advice over the telephone; while receiving advice via the Internet and Email received slightly higher preference ratings (13% for Internet and Email in this study, and 16% for internet and 19% for Email in the earlier study by Marshall et al. [18]).

Two other interesting comparisons are worth noting. The present data are again in agreement with Booth et al. [17], who reported that more women preferred to be active in a group compared to men (34% vs. 25%; women and men respectively, Booth et al. [17]). However, it is important to note that the proportion of women in the present study who reported they would like a group to be active with was much higher (60%) than the 34% reported for women by Booth et al. [17]. Second, and also in agreement with, Booth et al. [17], Indigenous men in the present investigation reported a strong preference for receiving advice from a GP or health professional. These data and preferences provide a platform on which to initiate further consultation with urban Indigenous communities in order that strategies to promote physical activity are appealing and relevant.

Data for this study were collected via self-report questionnaire. While questionnaires are an appropriate way to collect data from Indigenous adults [6,13], responses may be influenced by social desirability bias and the findings should be interpreted with this in mind. The present data were also limited to Indigenous adults living in suburban Brisbane, thus the findings may not be representative of and should not be generalised to other urban Indigenous groups.

In summary the main findings of this study suggest that Indigenous Australians living in Brisbane have similar levels of knowledge regarding the current PAG as do the broader Australian community. The primary exception related to knowledge that physical activity may be accumulated in ten minute bouts. In particular this needs to be better articulated to younger Indigenous people. Finally it appears that these and other suggestions relating to physical activity are most likely to be received if communicated via GPs or other health professionals (rather than via the telephone, email, books, videotapes etc.) and if programs that support activity groups are developed.

## Competing interests

The authors declare that they have no competing interests.

## Authors' contributions

All made substantial contributions to conception and design, analysis and interpretation of data. All authors were involved in drafting the manuscript and revised it critically for important intellectual content, and have read and approved the final manuscript.
